# Comparative Analysis of Digital Transcriptomics Between Pre- and Post-Treatment Samples of Patients with Locally Advanced Cervical Cancer: A Preliminary Study

**DOI:** 10.3390/cimb46110716

**Published:** 2024-10-28

**Authors:** Sunhwa Baek, Fabian Dominik Mairinger, Sabrina Borchert, Yue Zhao, Dominik Ratiu, Peter Konrad Mallmann, Henryk Pilch, Ka-Won Noh

**Affiliations:** 1 Department of Obstetrics and Gynecology, University Hospital Cologne and Medical Faculty, 50937 Cologne, Germany; 2 Institute for Pathology, University Hospital Essen, 45147 Essen, Germany; fabian.mairinger@uk-essen.de (F.D.M.); sabrina.borchert@rlk.uk-essen.de (S.B.); 3 Department of General, Visceral, Cancer and Transplantation Surgery, University Hospital Cologne and Medical Faculty, 50937 Cologne, Germany; yue.zhao@uk-koeln.de; 4 Institute for Pathology, University Hospital Cologne and Medical Faculty, 50937 Cologne, Germany

**Keywords:** cervical cancer, digital expression analysis, therapy resistance, precision oncology

## Abstract

Cervical cancer remains a leading cause of cancer-related deaths in women worldwide, with limited treatment options for advanced stages and therapy-resistant cases. Despite advances in treatment, the variability in the patient response to standard therapies underscores the need for molecular biomarkers to guide personalized treatment strategies. This study aimed to explore the transcriptomic changes associated with the therapeutic response in locally advanced cervical cancer, focusing on 770 immune-related genes. We employed a digital multiplexed gene expression analysis, comparing gene expression profiles between matching pre- and post-treatment samples. The results revealed the significant upregulation of C7 and EGR2 in the post-treatment samples, suggesting that enhanced immune activity is a key factor in therapeutic success. Conversely, IL17RB, S100A7, and SAA1 were upregulated in the pre-treatment samples, potentially indicating resistance mechanisms. Pathway enrichment analysis highlighted that the immune response and apoptosis pathways are crucial to post-treatment changes. These findings suggest that C7, EGR2, and IL17RB may serve as biomarkers for predicting therapeutic outcomes and could inform the development of more effective, individualized treatment strategies for cervical cancer. This study provides new insights into the molecular mechanisms underlying treatment response and resistance.

## 1. Introduction

Despite widespread screening programs and human papilloma virus (HPV) vaccines, cervical cancer is the fourth most common cancer in women, with a global incidence of 604,000 new cases and 342,000 deaths, displaying a particularly high burden in many low-income and middle-income countries [1]. It is most frequently diagnosed in women between the ages of 35 and 44, with the average age at diagnosis being 50, and is the second leading cause of cancer-related death for women aged 20 to 39 years [2]. In the United States, more than half of patients (54%) are diagnosed with advanced cervical cancer [2], which is even higher than in the developing countries [3]. In Germany, the age-standardized mortality rate for women has failed to decline since the early 2000s, suggesting that any further reduction in the rates may be increasingly difficult to achieve [4].

The response rate of chemoradiation, the standard of care for locally advanced cervical cancer in curative intention, varies strongly from patient to patient. The prognosis of advanced cervical cancer is poor, with a 5-year survival rate of 17% [3]. Targeting the vascular endothelial growth factor (VEGF) has shown benefits in the treatment of cervical cancer [5], and immunotherapy presents an additional rational approach for the treatment of cervical cancer given the molecular underpinnings of this HPV-related disease [6]. However, the overall response rate is still low. Moreover, the duration of stable disease is limited [5,6]. The therapeutic landscape highlights the need for rational combination therapies with the potential to provide improved responses and the identification of the molecularly defined subgroups who may benefit from each therapy regimen.

Although an increasing number of cervical cancer-related differentially expressed genes (DEGs) and pathways have been identified [7,8,9,10], the pathogenesis of cervical cancer still remains unclear in a considerable proportion of patients, especially for those with therapy resistance [11]. The cellular and molecular mechanisms underlying the development of resistance are multifactorial and include genetic and epigenetic alterations, cell detoxification, and abnormal drug efflux and accumulation [12]. Exploring the molecular mechanism of therapy resistance is pivotal in the development of strategies to overcome tumor resistance, improving cancer therapy efficacy [13].

This is the first study to our knowledge to utilize a digital multiplexed gene expression panel to detect and quantify the expression of a comprehensive set of immune-related genes in matching FFPE (Formalin-Fixed–Paraffin-Embedded) cervical cancer samples before and after treatment. By focusing on a panel of 770 genes that encompass crucial immune pathways, including innate and adaptive immune responses, cell migration, and immune checkpoint activity, we seek to gain a deeper understanding of the immunological landscape of cervical cancer in the frame of therapy. This study endeavors to identify specific immune markers and pathways that could inform the development of targeted therapies and improve the clinical outcomes for cervical cancer patients.

## 2. Materials and Methods

### 2.1. Study Cohort

Patients were identified retrospectively through a departmental database. Six women with a diagnosis of squamous cell carcinoma or adenocarcinoma of the cervix, who underwent a guideline-based therapy with matching pre- and post-treatment samples at the department of obstetrics and gynecology, University Hospital Cologne, between January 2016 and August 2021, were recruited (Figure S3). Four patients showed, despite treatment of care, a poor outcome with progression within 2 years (non-responder) and two showed an excellent outcome without progression or metastasis (responder). The FFPE tissues from each patient were collected at the time of first diagnosis (pre-therapeutic) and after the therapy (post-therapeutic). A retrospective analysis of the FFPE samples was approved for patients who provided informed consent at the time of initial diagnosis as part of the BioMaSOTA protocol, which is routinely collected during initial diagnosis (protocol code: 22-1475). To validate our candidate genes, Cervical Squamous Cell Carcinoma and Endocervical Adenocarcinoma dataset (N = 310) that was available from The Cancer Genome Atlas Program (TCGA) database was used [14,15,16].

### 2.2. Macrodissection and RNA Isolation

Six to ten 10 µm sections were cut from the FFPE tissue blocks for RNA extraction. H&E-stained slides with the markings of a tumor were provided as a reference, and this was performed by a trained pathologist (A.S.). As there were no differences in tumor purities between the two groups, no further correction was performed (Figure S1A). RNA was extracted as previously described [17]. Briefly, the samples were directly scraped off from unstained sections, then further prepared using the Maxwell RSC RNA FFPE Kit with the inclusion of a DNAse digestion step according to the manufacturer’s instructions (Promega, Madison, WI, USA). RNA was quantified using NanoDrop Spectrophotometer (Thermo Fisher Scientific, Waltham, MA, USA).

### 2.3. Digital Gene Expression Analysis

Digital gene expression analysis was conducted using the NanoString nCounter^®^ platform (NanoString Technologies, Inc., Seattle, WA, USA) with the appertaining NanoString nCounter^®^ PanCancer Immune Profiling Panel, which includes 770 genes for a comprehensive assessment of immune pathways. This includes the activation of innate and adaptive immune responses, cell migration, and immune checkpoint activity, along with 30 reference genes for normalization.

The probes were hybridized to 100 ng of total RNA for 20 h at 65 °C, then processed on the nCounter^®^ PrepStation. Post-hybridization processing was conducted using the nCounter^®^ Max/Flex System with a high-sensitivity protocol. The cartridge was subsequently scanned and analyzed on the DigitalAnalyzer at 555 fields of view (FOV).

Data processing was conducted using R statistical software (version 4.0.3) [18]. The initial normalization of raw NanoString counts (Supplementary File S1) was performed by adjusting for technical variability, using the mean counts plus two standard deviations from the negative controls of the CodeSet. Following this, biological normalization was achieved using the included mRNA reference genes. Counts with a *p*-value greater than 0.05, determined by an one-sided *t*-test against negative controls plus two standard deviations, were considered not expressed to reduce background noise.

### 2.4. Statistical Analysis

Statistical and graphical analyses were also performed within the R statistical programming environment (v. 4.0.3) [18]. The Shapiro–Wilk test was applied to test for the normal distribution of the data [19]. For dichotomous variables, either the Wilcoxon Mann–Whitney rank sum test (non-parametric) or the two-sided Student *t*-test (parametric) was applied [20]. For ordinal variables with more than two groups, either the Kruskal–Wallis test (non-parametric) or ANOVA (parametric) was used to detect group differences. Double dichotomous contingency tables were analyzed using Fisher’s exact test. To test the dependency of ranked parameters with more than two groups, the Pearson’s Chi-squared test was used. The correlations between metric variables were tested by using the Spearman’s rank correlation test as well as Pearson’s product moment correlation coefficient for linear modeling.

The pathway analysis was based on the KEGG database (Kyoto Encyclopaedia of Genes and Genomes) and was performed using the “pathview” package in R. Differences were specified by −log2-fold changes between the means (parametric) or medians (non-parametric) of the compared groups.

Gene set enrichment analysis (GSEA) was performed using the WEB-based Gene SeT AnaLysis Toolkit (WebGestalt) website [21]. In order to investigate certain signaling pathways, the differential gene expression analysis was visualized on molecular network maps. These maps were provided by KEGG [22].

In order to overcome the problem of repeated statistical testing, *p*-values were corrected by utilizing the false discovery rate (FDR). Results were considered significant at *p* < 0.05 after adjustment.

To identify individual patient transcription differences, in silico analysis tools were used. For creating a hierarchically clustered heatmap, SRplot was used. To further analyze the DEGs in a volcano plot, discrimination was achieved by applying a significance threshold of at least *p* < 0.05 and a minimum change of log2 fold changes > 0.5.

The adaption of profiles was modeled by the supervised machine learning tool conditional inference trees (CITree), as implemented in the “party” library of R [23], using leave-one-out cross-validation. The CITree is a non-parametric class of regression trees to a non-parametric class of tree-structured regression models that embed a conditional inference procedure applicable to all kinds of regression problems, including nominal, ordinal, numeric, and censored as well as multivariate response variables and arbitrary measurement scales of the covariate [23].

## 3. Results

### 3.1. Patient Characteristics

The median age at diagnosis was 42.5 years (34–55 years). The main clinicopathological characteristics are listed in Table 1 and the therapies of each patient from initial diagnosis until the post-treatment samples were obtained are reported separately (Figure S4). The time from the end of primary treatment to the detection of progression or metastasis was defined as progression-free survival (PFS). The time from the end of primary treatment to death was defined as overall survival (OS). Patients who developed rapid recurrences or metastasis after receiving the guideline-based standard of care were defined as non-responders (C1, 2, 5, and 6), two of whom died. Two patients with FIGO stage IIB (C3 and 4) showed an excellent prognosis after system therapy and surgery with radical hysterectomy and pelvic lymph node dissection, and were defined as responders.

### 3.2. Transcriptomic Changes in Pre- and Post-Treatment Cervical Carcinoma Samples

**Figure 1:**
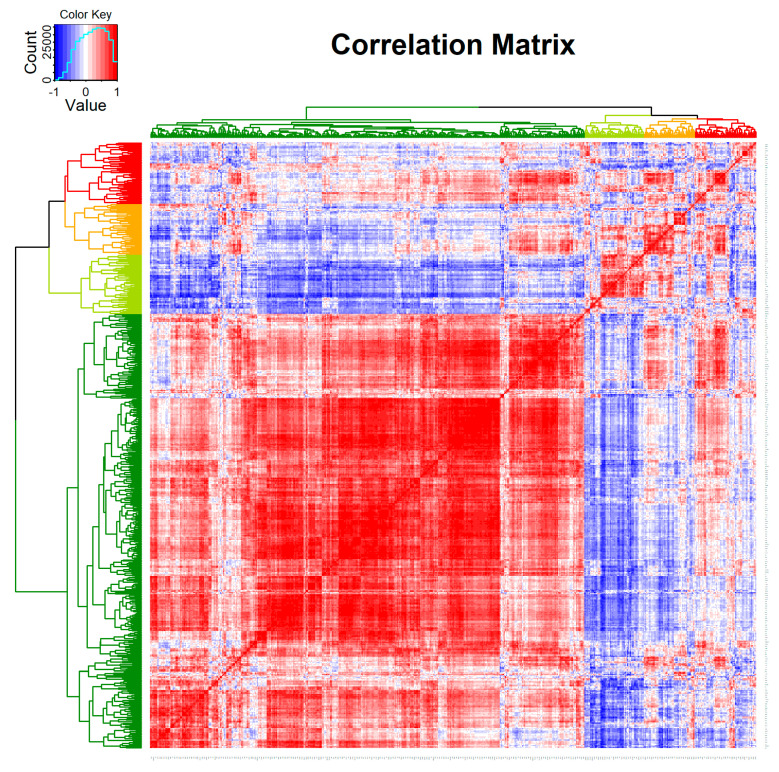
**Correlation matrix of the expression of 770 genes in the matching pre- and post-therapeutic cervical cancer samples.** Unsupervised clustering between genes indicates four potential clusters in the analyzed samples of six patients (Red: correlation = 1; Blue: correlation = −1).

After the initial quality control and data processing of the matching patient samples, 770 genes remained for further analysis. The correlation matrix of the expression levels of 770 genes in the pre- and post-treatment cervical cancer samples provides a comprehensive overview of the transcriptomic changes (Figure 1). The heatmap shows distinct clusters based on the gene expression profiles. The unsupervised clustering reveals four primary clusters, indicated by the dendrogram on the left.

**Figure 2:**
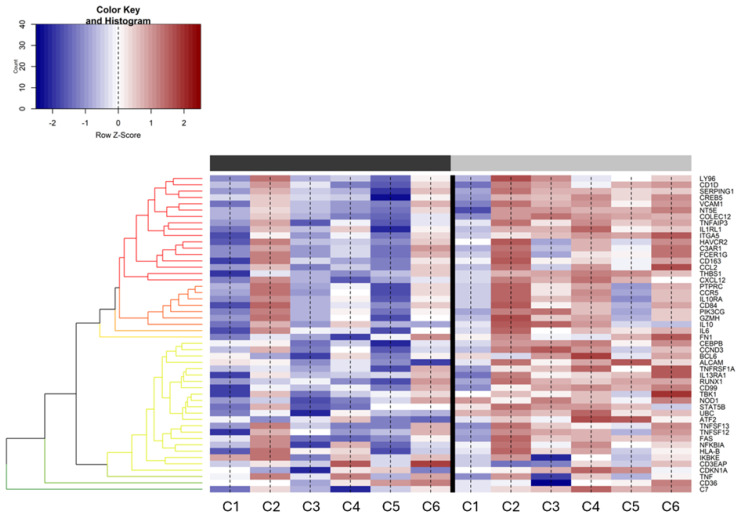
**Differential expression of genes (DEGs) between pre- and post-treatment samples.** The heatmap displays a range of expression levels, with a color key from red (upregulation) to blue (downregulation). The column bar represents the time point of sample generation (black: pretreatment, grey: post-treatment). All referenced genes are displayed as DE between the two conditions (false discovery rate (FDR) < 0.05). Counts are given as log (counts per million (CPM)).

Differential gene expression analysis was conducted to assess any significant changes in gene expression between the pre- and post-treatment samples. The transition from predominantly blue in the pre-treatment samples to red in the post-treatment samples indicates a substantial upregulation of certain genes post-treatment (Figure 2). Notably, genes related to the immune response show a marked increase in expression in the post-treatment samples, suggesting an enhanced immune reaction following treatment.

**Figure 3:**
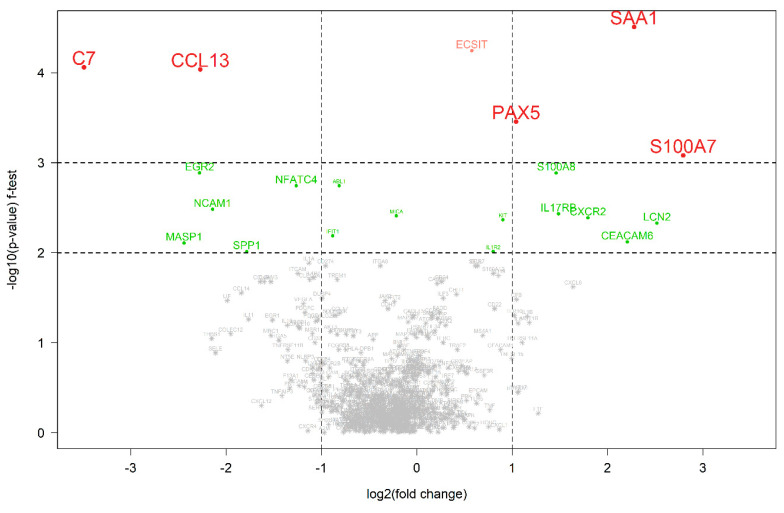
**Differentially expressed genes between pre- and post-therapeutic cervical carcinoma samples.** A volcano plot with the statistical significance of the expression level of 770 genes between pre- and post-therapeutic pairs measured using the f-test. A negative log2-fold change indicates that the gene is upregulated in the post-therapeutic samples in comparison to the pre-therapeutic samples. Colored genes represent those that were significantly differentially expressed in comparison to the other group.

A further analysis to highlight significant alterations in gene expression was conducted using a volcano plot (Figure 3). Notably, the plot identifies several genes with substantial changes, suggesting their potential role as biomarkers of the therapeutic response. The significant upregulation of the CCL13 (C-C motif chemokine ligand 13) and C7 (complement C7) genes in the post-therapy samples indicates their involvement in the therapeutic effect, whereas the PAX5 (paired box 5), SAA1 (serum amyloid A1) and S100A7 (S100 calcium binding protein A7) genes upregulated in pre-therapy may be associated with the untreated disease state.

### 3.3. Pathway Enrichment Analysis

**Figure 4:**
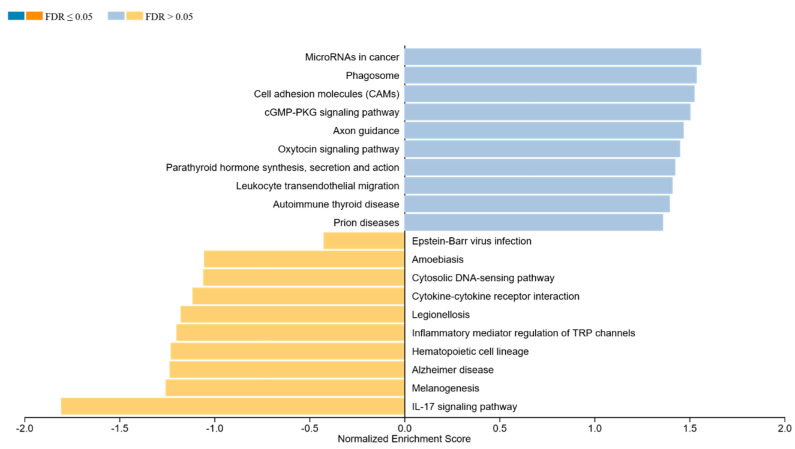
**Gene set enrichment analysis (GSEA) between the pre- and post-therapeutic cervical carcinoma samples.** Blue bars indicate pathways enriched in the pre-therapy group and yellow indicates the post-therapy group.

Figure 4 presents the GSEA results, revealing the pathways significantly enriched in either the pre- or post-therapeutic groups. Pathways enriched in the pre-therapy group are indicated by blue bars, while those enriched post-therapy are shown in yellow. The analysis indicates distinct biological processes and pathways that are differentially regulated following treatment. For instance, the pathways involved in cell proliferation and survival may be downregulated post-therapy, whereas those related to the immune response and apoptosis could be upregulated, reflecting the therapeutic impact on tumor biology. An overview of all genes and the specific pathways is given in Supplementary File S2.

### 3.4. Predictive Value of Significantly Differentiated Genes for Response

**Figure 5:**
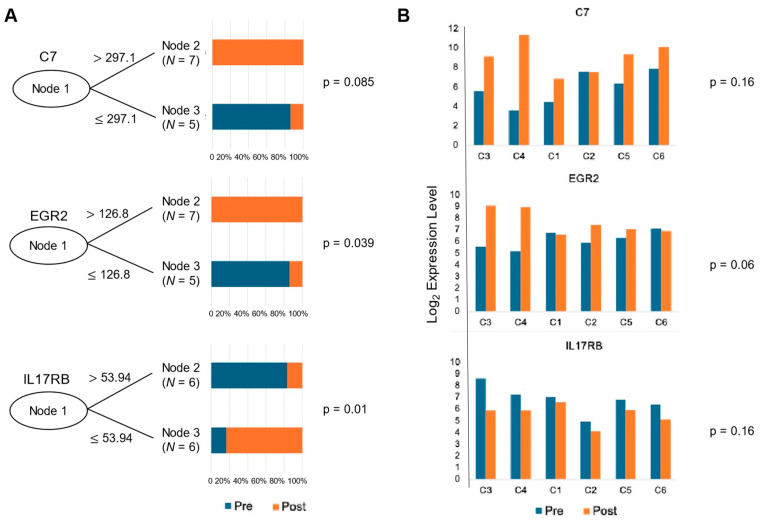
Classifier result of significantly differentiated genes in cervical carcinoma samples. (**A**) A conditional inference tree (CIT)-based classifier was used to determine the appropriate cut-off values in order to determine whether certain genes can accurately classify pre- and post-treatment samples. (**B**) Bar plots of the candidate genes illustrating the change in gene expression levels between pre- and post-treatment in each patient (C1, C2, C5, C6: non-responder, C3, C4: responder). P-values were generated using the Kruskal–Wallis test (*p* = 0.16, 0.06, 0.16, respectively).

The CIT-based classifier was employed to identify key genes that can distinguish between pre- and post-therapeutic samples, as shown in Figure 5A. This classifier determined the optimal cut-off values for the gene expression levels. High expression levels of C7 and EGR2 (early growth response 2) were found to be significant predictors of post-treatment samples (*p* = 0.085 and 0.039, respectively), while IL17RB (interleukin 17 receptor B) expression was a predictor of pre-treatment samples (*p* = 0.01). The bar plots display the proportion of pre- and post-therapeutic samples correctly classified based on these cut-offs, underscoring the potential utility of these genes as biomarkers of the therapeutic response.

The bar plots in Figure 5B illustrate the gene expression levels of C7, EGR2, and IL17RB, categorized by their response to therapy (two responders on the left-side and the non-responders on the right). The Kruskal–Wallis test *p*-values (*p* = 0.16, 0.064, and 0.16, respectively) indicate varying degrees of statistical significance in the differences in gene expression between the responders and non-responders. This suggests that while some genes may not significantly differentiate between these groups, others, like EGR2, show promise as potential indicators of therapeutic efficacy.

As these findings highlight the complex relationship between gene expression and treatment response, we decided to further validate this in a larger cohort using the TCGA Cervical Squamous Cell Carcinoma and Endocervical Adenocarcinoma dataset. There, we were able to find tumor response data from 49 samples. A complete response and partial response were grouped into the responder group (N = 44), while a stable response and progressive disease were grouped into the non-responder group (N = 5). The RNA levels for each candidate genes can be found in Figure S1B–F. For the genes C7, EGR2, and S100A7, similar expression patterns could be seen in the validation cohort as well (Figure S1B,C,F). Although IL17RB showed a slightly higher median expression in the responder group, the expression of IL17RA, a paralog of IL17RB, was higher in the non-responder group (Figure S1D,E).

## 4. Discussion

The present study is the first, to our knowledge, to utilize a digital gene expression analysis for the comprehensive quantification of immune-related genes in matching FFPE cervical cancer samples, along with pre- and post-therapeutic changes. We sought to identify specific immune markers and pathways that could inform the development of targeted therapies and improve the clinical outcomes for cervical cancer patients. The findings from our analyses collectively indicate significant transcriptomic reprogramming in cervical cancer tissues in response to treatment.

The differential gene expression analysis revealed the significant upregulation of immune-related genes post-therapy, underscoring the role of the immune system in the therapeutic response. Notably, genes such as C7 and EGR2 were shown to be upregulated post-treatment for those who responded well to therapy (Figure 3 and Figure 5). C7 is a terminal component of complement activation, which plays a pivotal role in innate immunity [24], and it is also known to be a potential tumor suppressor [25,26]. The downregulation of C7 was associated with poor differentiation and subsequently a poor prognosis for certain cancers such as ovarian, gastric and prostate cancer and NSCLC [25,26,27]. EGR2 is a zinc-finger transcription factor of the early growth response gene (EGR) family [28], which has critical functions in the development of natural killer T cells and self-tolerance [29,30]. EGR2 also plays a key role in the PTEN-induced apoptotic pathway [31]. The post-therapeutic upregulation of C7 and EGR2 likely led to an enhancement in immune responses and apoptosis, thereby contributing to the therapeutic effect.

Genes such as SAA, S100A7 and IL17RB could be identified as pre-therapeutic upregulated genes (Figure 3). As a positive acute phase protein, SAA is produced primarily by the liver in response to trauma, infection, inflammation, and neoplastic stimuli [32]. Recent data suggest that SAA has an impact on carcinogenesis by activating the transcriptional factor nuclear factor kappa-B [33,34] and inducing the expression of matrix metalloproteinases [35,36] involving cell proliferation and migration. The high expression of SAA in cervical cancer was reported [37]. The S100 family of proteins is involved in the regulation of cellular processes such as cell cycle progression and differentiation, and many S100s may also promote cancer progression through specific roles in cell survival pathways [38]. Tian et al. suggested that S100A7 may promote the migration and invasion of cervical cancer cells by epithelial–mesenchymal transition and exosome secretion [39]. The interleukin-17 (IL-17) superfamily is known to play an essential role in the development of inflammatory diseases and some types of cancers [40]. Recent studies showed that the IL17B/IL17RB cascade promotes antiapoptosis and tumorigenesis in breast cancer [41,42] and facilitates invasion, vasculogenic endothelial cells and the macrophage recruitment of pancreatic cancer cells [43]. For cervical cancer, it has been assumed that T helper 17 (Th17) cells may be involved in the proliferation of cervical cancer cells, since a high expression of IL-17 has been identified in the cervical mucosa of patients [44]. The immune response of Th17 to persistent HPV infection in the genital tract triggers chronic inflammation with the prolonged production of IL-17 and other pro-inflammatory cytokines, making a favorable environment for tumorigenesis [45]. Punt et al. showed that IL-17 promotes tumor growth, and that IL-17+ cells are independently associated with poor prognosis in cervical cancer [46]. Consistent with these findings, our data show that genes upregulated in pre-treatment are primarily involved in tumor proliferation, invasion and survival pathways, suggesting potential targets for therapeutic intervention. Notably, IL17RB was shown to be significantly downregulated in responders during the course of the therapy, whereas no relevant change in the expression of IL17RB was observed for those who did not respond to the therapy (Figure 5).

The pathway enrichment analysis further supports these observations (Figure 4). In post-treatment, there is a notable enrichment of pathways associated with immune response and apoptosis, implying a shift towards tumor regression and the immune-mediated destruction of cancer cells [47,48,49,50] (Figure S2). These findings align with the mechanisms of action for many cancer therapies, which aim to inhibit tumor growth and enhance immune surveillance, highlighting the importance of targeting specific biological processes to improve therapeutic outcomes.

C7, EGR2, and IL17RB were identified as key genes to predict treatment responses (Figure 5). High expression levels of C7 and EGR2 were associated with the post-treatment samples, while IL17RB was linked to the pre-treatment samples. This ability to differentiate between pre- and post-therapeutic samples underscores the potential of these genes as biomarkers for monitoring treatment efficacy. The known biological functions of these genes support their predictive value. C7 is part of the complement system, which enhances the body’s ability to clear pathogens and damaged cells, promoting an inflammatory response. In cervical cancer, the upregulation of C7 can suggest increased complement activation, leading to membrane attack complex (MAC) formation and the recruitment of immune cells. Studies suggest that the activation of the complement cascade contributes to the killing of tumor cells when combined with immunotherapy. For example, immunotherapies like checkpoint inhibitors can boost T-cell activity, which synergizes with complement-mediated lysis [51]. EGR2 is a transcription factor involved in the regulation of immune responses, particularly in T-cell development and function. EGR2 is a transcription factor involved in the regulation of immune responses, particularly in T-cell development and function. Moreover, it may modulate cytokine production, influencing immune-mediated tumor suppression. EGR2 may enhance the tumor microenvironment’s responsiveness to treatment by improving T-cell survival and effector function [52]. In cervical cancer, the upregulation of EGR2 may enhance the anti-tumor immune response by promoting the differentiation of T-cells into a more cytotoxic phenotype, which is essential for eliminating cancer cells during therapies such as chemoradiation and immunotherapy. This result was further investigated in responders and non-responders to therapy to assess its potential as a marker of the therapeutic response. Although the Kruskal–Wallis test results showed varying degrees of statistical significance, the trend suggests higher expression levels of C7 and EGR2 in responders compared to non-responders. This supports the hypothesis that effective therapy induces immune activation and apoptosis [25,26,31]. While our dataset did not specifically resolve immune cell subsets, the increased expression of genes involved in immune cell recruitment and activation, such as CCL13, suggests that immune cell infiltration likely contributes to the observed response (Figure S2C). The lack of a significant difference for IL17RB may indicate a more complex role, requiring further investigation [41,42,46]. Overall, these findings suggest that monitoring the expression of these genes could help predict patients’ response to therapy and tailor treatment strategies accordingly.

The results of this study have several important implications for the management of cervical cancer. Identifying differentially expressed genes and enriched pathways provides potential targets for therapeutic intervention and biomarkers for monitoring treatment response. For example, neoadjuvant chemotherapy (NACT) followed by radical surgery has been considered as an alternative approach to improve disease control and reduce toxicity. Although many studies have demonstrated feasible outcomes for NACT and surgery regarding response rates and toxicity, its impact on overall survival remains unproven [53,54,55]. In our study, two patients treated with NACT achieved excellent PFS, whereas one patient developed rapid disease progression despite receiving the same treatment. For patients without access to chemoradiation [56] or those seeking fertility preservation [57], NACT followed by tailored surgical intervention may offer a viable alternative if they present the corresponding biomarker. The predictive value of C7, EGR2, and IL17RB suggests these genes could be used to develop diagnostic tools for assessing treatment efficacy in clinical settings.

While our study offers valuable preliminary insights into the transcriptomic changes associated with therapy in patients with locally advanced cervical cancer, we recognize several limitations that may impact the interpretation and generalizability of our findings. First, the limited sample size and preliminary nature of our research may affect the robustness of our conclusions, reducing their broader applicability. Furthermore, the potential influence of confounding variables such as tumor properties, alongside treatment protocol variations and individual patient characteristics, likely contribute to the observed variability in gene expressions and clinical outcomes. These factors underline the importance of approaching our findings with caution. Another potential limitation of our study is the use of digital multiplexed gene expression analysis. The results are dependent on the pre-selected gene panel comprising 770 genes focusing on specific cancer pathways and related immune regulation. In addition, using this technique may have led to the underestimation of genes expressed at very low levels. Some inherent biases may lie in the normalization procedures as well as the technical variability, which may have affected the final results. In the panel, there are appropriate controls as well as housekeeping genes employed to mitigate these issues. However, despite these limitations, we believe that our study lays a valuable foundation for future research, highlighting the need for larger and more comprehensive studies to elucidate the transcriptomic responses to therapy in this patient population. Future studies should focus on validating these findings in larger, independent matched cohorts to confirm their utility as biomarkers. While the present study focused on the transcriptomic changes in response to therapy, further analysis on a longitudinal timeline in relation to clinical outcomes could be conceptualized after the validation of methods and the confinement of DEGs. In this way, precision medicine approaches could be developed to stratify patients by their likelihood to respond to treatment, allowing for individualized therapy regimens that improve efficacy and minimize side effects. This would also enable the identification of early biomarkers of response or resistance, as well as the long-term changes associated with sustained remission or recurrence. Although we have investigated this using the TCGA dataset, we were only able to observe the similar patterns that were observed using our cohort without stastical significance. This may be attributed to the unmatched nature of the patient cohort and the small number of non-responders included in the dataset. Functional studies are also needed to elucidate the precise roles of these genes in mediating the therapeutic response. Investigating the underlying mechanisms through which these genes influence tumor biology could provide insights into new therapeutic strategies. Moreover, integrating gene expression data with other molecular and clinical parameters could enhance the predictive power of these transcriptomic changes and lead to more personalized treatment approaches.

In conclusion, this study highlights the potential of transcriptome expression profiling and pathway analysis in understanding the molecular effects of therapy and identifying biomarkers for cervical cancer. These findings provide a foundation for further research aimed at improving treatment outcomes and developing targeted therapies for this malignancy.

## Figures and Tables

**Table 1:** Clinicopathological characteristics.

Patient ID	C1	C2	C3	C4	C5	C6
Median Age in yeas	34	55	36	52	46	39
Date of first Diagnosis	Jan 2019	Jan 2018	Jan 2016	Feb 2018	Aug 2021	May 2018
FIGO stage 2018	IB3	IB1	IIB	IIB	IIIB	IIB
Histological type	SCC	AC	AC	SCC	SCC	SCC
Grading	G3	G3	G1	G3	n.d.	G2
Lymphovascular invasion	pos.	neg.	neg.	neg.	pos.	pos.
PFS	17	17	n.d.	n.d.	1	18
OS	59	n.d.	n.d.	n.d.	11	n.d.

SCC: Squamous cell carcinoma, AC: Adenocarcinoma, pos.: positive, neg.: negative, n.d.: not determined (patients are still alive or no progression has been detected to date).

## Data Availability

The original data presented in the study are included in the Supplementary Materials. Further inquiries can be directed to the corresponding author.

## Supplementary Materials

The following supporting information can be downloaded at: https://www.mdpi.com/article/10.3390/cimb46110716/s1.
